# Polyethylene glycol recombinant human growth hormone in Chinese prepubertal slow-growing short children: doses reported in a multicenter real-world study

**DOI:** 10.1186/s12902-022-01101-8

**Published:** 2022-08-09

**Authors:** Jiajia Chen, Yan Zhong, Haiyan Wei, Shaoke Chen, Zhe Su, Lijun Liu, Liyang Liang, Ping Lu, Linqi Chen, Ruimin Chen, Shining Ni, Xinli Wang, Li Li, Yunfeng Wang, Xu Xu, Yanfeng Xiao, Hui Yao, Geli Liu, Runming Jin, Bingyan Cao, Di Wu, Chang Su, Wenjing Li, Miao Qin, Xiaoqiao Li, Xiaoping Luo, Chunxiu Gong

**Affiliations:** 1 Department of Endocrine and Genetics and Metabolism, Beijing Children’s Hospital, Capital Medical University, National Centre for Children’s Health, No.56 Nanlishi Road, Xicheng District, 100045 Beijing, China; 2 Department of Child Health Care, Hunan Children’s Hospital, Changsha, 410007 China; 3 Department of Endocrinology and Metabolism, Genetics, Henan Children’s Hospital (Children’s Hospital Affiliated to Zhengzhou University), Changsha, 450018 China; 4 Department of Genetics and Metabolism, The Second Affiliated Hospital of Guangxi Medical University, Nanning, 530005 China; 5 Department of Endocrinology, Shenzhen Children’s Hospital, Shenzhen, 518038 China; 6 Department of Endocrinology, Genetics, and Metabolism, Hebei Children’s Hospital, Shijiazhuang, 050031 China; 7 Department of Pediatrics, Sun Yat-Sen Memorial Hospital, Sun Yat-Sen University, Guangzhou, 510120 China; 8 Department of Pediatrics, The First Affiliated Hospital of Kunming Medical University, Kunming, 650032 China; 9 Depatment of Endocrinology, Children’s Hospital of Soochow University, Suzhou, 215025 China; 10 Department of Endocrinology, Geneticsand Metabolism, Fuzhou Children’s Hospital of Fujian Medical University, Fuzhou, 350005 China; 11 Department of Endocrinology, Children’s Hospital of Nanjing Medical University, Nanjing, 210008 China; 12 Department of Pediatrics, Peking University Third Hospital, Beijing, 100191 China; 13 Department of Pediatrics, The First People’s Hospital of Yunnan Province, Kunming, 650032 China; 14 Department of Pediatrics, China-Japan Friendship Hospital, Beijing, 100029 China; 15 Department of Endocrinology, Wuxi Children′s Hospital, Wuxi, 214023 China; 16 Department of Pediatrics, The 2nd Affiliated Hospital of Medical College of Xi’an Jiaotong University, Xi’an, 710004 China; 17 Department of Endocrinology and Metabolism, Wuhan Children’s Hospital, Tongji Medical College, Huazhong University of Science and Technology, Wuhan, 430015 China; 18 Department of Pediatrics, Tianjin Medical University General Hospital, Tianjin, 300052 China; 19 Department of Pediatrics, Union Hospital, Tongji Medical College, Huazhong University of Science and Technology, Wuhan, 430022 China; 20 Department of Pediatrics, Tongji Hospital, Tongji Medical College, Huazhong University of Science and Technology, Wuhan, 430030 China

**Keywords:** Short stature, Children, Polyethylene glycol recombinant human growth hormone, Multicenter, Real-world study

## Abstract

**Background:**

To evaluate the effectiveness of individualized-dose polyethylene glycol recombinant human growth hormone (PEG-rhGH) for short stature.

**Methods:**

This real-world study enrolled children with short stature in 19 hospitals throughout China. They were treated with PEG-rhGH for 6 months. The starting dosage ranged from 0.10 to 0.20 mg/kg/week. The primary outcome was the change in height standard deviation score (ΔHt SDS).

**Results:**

Five hundred and ten patients were included and grouped based on dosage as A (0.10–0.14 mg/kg/week), B (0.15–0.16 mg/kg/week), C (0.17–0.19 mg/kg/week), and D (0.20 mg/kg/week). The mean 6-month ΔHt SDS for the total cohort was 0.49 ± 0.27, and the means differed among the four dose groups (*P* = 0.002). The ΔHt SDS was lower in group A than in groups B (LSM difference [95%CI], -0.09 [-0.17, -0.01]), C (LSM difference [95%CI], -0.10 [-0.18, -0.02]), and D (LSM difference [95%CI], -0.13 [-0.21, -0.05]) after adjusting baseline covariates. There were no significant differences among groups B, C, and D. When the baseline IGF-1 was < -2 SDS or > 0 SDS, the △Ht SDS was not different among the four groups (*P* = 0.931 and *P* = 0.400). In children with baseline IGF-1 SDS of -2 ~ 0 SDS, a higher dosage was associated with a better treatment effect (*P* = 0.003), and the △Ht SDS was lower in older children than in younger ones (*P* < 0.001).

**Conclusions:**

PEG-rhGH could effectively increase height in prepubertal short children. When the baseline IGF-1 was < -2 SDS, 0.10 mg/kg/week could be a starting dose. In other IGF-1 statuses, 0.15–0.20 mg/kg/week might be preferred.

**Trial registration:**

ClinicalTrials.gov: NCT03249480, retrospectively registered.

**Supplementary Information:**

The online version contains supplementary material available at 10.1186/s12902-022-01101-8.

## Background

Short stature is generally defined as height < -2 standard deviations for the population mean for age and sex or lower than the 3^rd^ percentile of the growth curve of children of the same age and sex [1, 2]. It is the most common reason for referral to pediatric endocrinologists. Isolated short stature can be idiopathic or due to growth hormone deficiency (GHD). The diagnosis of GHD in childhood is complex. Idiopathic short stature (ISS) can be confused for GHD, and partial GHD is particularly difficult to distinguish from ISS [3–6].

Recombinant human growth hormone (rhGH) is the main treatment to normalize adult height and avoid extreme shortness in children and adolescents [2–4, 7]. Exogenous GH increases insulin-like growth factor (IGF-1), which promotes the growth of long bones [8]. rhGH was approved to treat short stature caused by many diseases, including GHD and ISS. The starting dose of rhGH and dose adjustments are mainly based on weight or body surface area and growth response. An individualized dosage of rhGH is required and aims for the lowest dose at which there is an appropriate response in height velocity. For patients with GHD, the treatment effect is often obtained even at a relatively low dosage of GH. For short stature caused by other diseases, the dose of rhGH is relatively large [9].

The currently used rhGH are mainly short-acting formulations, and a daily injection given at bedtime is required to mimic the normal daily profile of increased GH production at night [10, 11]. The long-term use of rhGH can lead to injection pain and poor compliance [12–14]. Polyethylene glycol recombinant human growth hormone (PEG-rhGH) is a long-acting formulation injected once per week [13–17]. The PEGylation of rhGH confers long-acting effects due to higher protein stability, reduced non-specific absorption and antigenicity, reduced renal clearance, and extended elimination half-life [15–17]. Pharmacokinetics curves showed that PEG-rhGH at 0.10–0.40 mg/kg persists in circulation for 192 h after injection [18]. Previous phase 1, 2, and 3 clinical ﻿﻿trials demonstrated that compared with short-acting GH preparations, PEG-rhGH injection (Jintrolong®) at 0.20 mg/kg/week is effective and safe in treating children with GHD [18–20] and is probably not inferior to daily rhGH [19]. Hou et al. [20] reported a similar increase in IGF-1 between PEG-rhGH 0.20 mg/kg/week and rhGH 0.20 mg/kg/week.

PEG-rhGH was approved in January 2014 in China to treat GHD-related short stature in children. Long-acting GH formulations might improve compliance and consequently improve efficacy. Although PEG-rhGH has promising prospects in clinical practice, post-marketing data are lacking. In addition, the clinical trials only studied two dosages, limiting the generalizability of the findings, and there is an urgent need for real-world results [21, 22].

Therefore, this study aimed to evaluate the effectiveness of individualized-dose PEG-rhGH in children with short stature in a large-scale, prospective, real-world study. The results could reveal the influencing factors of the clinical effectiveness of PEG-rhGH for short stature children in China and examine the initial treatment dosage of PEGylated GH based on the IGF-1 levels at baseline.

## Methods

### Study design and participants

This multicenter real-world study enrolled children with short stature in 19 hospitals throughout China between January 2015 and December 2017. This study was approved by the Ethics Committees of the Beijing Children’s Hospital, Capital Medical University ([2015]-Y-001-C), and all other participating centers. The study was performed in accordance with the relevant guidelines and regulations. Written informed consents were obtained from all the children and their guardians. The participants were recruited according the criteria of the clinical trial (ClinicalTrials.gov: NCT03249480, 15/08/2017). However, partial participants did not strictly follow the original design of the clinical trial and were treated clinically. Then the physicians and the guardians decided the treatment together as in the real-world clinical practice.

The inclusion criteria were 1) met the diagnosis criteria of short stature, which included: i) absolute height lower than the 3^rd^ percentile of the growth curve of the Chinese children population of the same age and sex, according to the height data reported by the 2005 national survey in China [23], ii) growth velocity (GV) of ≤ 5.0 cm/year, iii) peak plasma GH concentration < 10.0 ng/mL in two stimulation tests with different mechanisms within 1 year before starting treatment, and iv) bone age (BA) ≤ 9 years for girls, and ≤ 10 years for boys, 2) prepubertal status (Tanner stage I), 3) ≥ 3 years old, and 4) did not receive GH treatment within the last 6 months.

The exclusion criteria were 1) liver and/or renal insufficiency with alanine transaminase (ALT) > twofold the upper limit of normal (ULN) or creatinine (Cr) > ULN), 2) positive hepatitis B virus examination including HBcAb, HBsAg, and HBeAg, 3) known highly allergic constitution or allergic to the study drug, 4) diabetes, 5) severe cardiopulmonary diseases, hematological diseases, malignant tumors, systemic infection, immune hypofunction, or psychiatric diseases, 6) other abnormalities in growth and development, including Turner syndrome, Laron syndrome, and GH receptor deficiency, 7) participation in other clinical trials of drugs within the last 3 months, or 8) congenital skeletal dysplasia.

### Treatment

All children were treated with PEG-rhGH injection (Jintrolong®; GeneScience Pharmaceuticals, Changchun, China) once per week for 6 months. The individualized dosage was determined by the physicians based on clinical experience, ranging from 0.10 to 0.20 mg/kg/week. The dosages of PEG-rhGH were not adjusted during the treatment period.

### Data collection

The clinical data, including sex, age, body temperature, heart rate, respiration, blood pressure, height, weight, and Tanner stage, were collected. The laboratory examination included blood routine test, liver and renal functions, electrolytes, blood lipid, thyroid functions, cortisol, adrenocorticotropic hormone (ACTH), GH stimulation test, insulin-like growth factors-1 (IGF-1), insulin-like growth factor binding protein-3 (IGFBP-3), anti-hGH antibody, and chromosome karyotypes (for female patients). The imaging examinations included X-ray for BA, anteroposterior and lateral X-ray of the whole spine, and pituitary magnetic resonance imaging (MRI). Electrocardiogram was also monitored.

The height and body weight of the children were measured by specially assigned investigators in each study center, using the specially assigned weighing machine and height-measuring device. The Atlas of Greulich and Pyle was used for the assessment of BA. IGF-1 and IGFBP-3 levels were measured by the chemiluminescence method (Immulite 2000 analyzer; Siemens, Erlangen, Germany). The children were carefully followed up and examined before, during, and after treatment, and the indicators of effectiveness and safety were monitored and recorded. No dietary restrictions were applied in this study, and the children were ensured with sufficient nutrition intake to maintain normal nutritional status. The children were required to avoid exercise for one day before screening and follow-up examinations.

### Follow-up and outcomes

During the 26 weeks of intervention, the children were examined four times, i.e., at baseline and 4, 13, and 26 weeks of treatment.

The primary outcome was the height standard deviation score (Ht SDS) by the chronological age (CA) at the end of 26 weeks of treatment. △Ht SDS_CA_ was defined as the Ht SDS at 26^th^ week – height SDS at baseline. Height SDS = (height at the assessment – the average height of normal children of the same age and sex) / (standard deviation of height of normal children of the same age and sex).

The secondary outcome was the blood IGF-1 standard deviation score (IGF-1 SDS). Blood IGF-1 SDS = (actual IGF-1 level – median IGF-1 level of normal children of the same age and sex) / (the standard deviation of IGF-1level of normal children of the same age and sex).

The safety indicators included tolerability indicators, thyroid function, liver, and renal functions, blood glucose, glucose metabolism indicators, blood lipid, anti-hGH antibody positive, and general manifestations. The tolerability indicators were injection reactions of the local skin, allergy, limb pain, joint pain, gynecomastia, benign intracranial hypertension, intermittent claudication, slipped capital femoral epiphysis, scoliosis, peripheral edema, etc.

### Statistical analysis

The continuous data were tested for normal distribution using the Kolmogorov–Smirnov test. Continuous data with a normal distribution were described as means ± standard deviations and tested using analysis of variance (ANOVA) and the least significant difference (LSD) post hoc test for intergroup comparison and paired t-test for intragroup comparison. Continuous data without normal distribution were described as median (IQR, interquartile range) and tested using the Kruskal–Wallis non-parametric test. Categorical data were described as n (%) and tested using the chi-square test or Fisher’s exact test. The significant (*P* < 0.05) variables in the univariable analyses were included in a multivariable analysis. Furthermore, the covariance analysis model was applied to assess the 6-month ΔHt SDS, with the dosage being the fixed effect and with the other baseline characteristics (such as IGF-1 SDS, sex, and baseline age) as covariates. The least-squares mean (LSM) estimates of 6-month ΔHt SDS in each dosage group were calculated. The differences in LSM among the different dosage groups with 95% CI were shown. The statistical analyses were two-sided, and *P*-values < 0.05 were considered statistically significant.

## Results

### Characteristics of the children

A total of 699 children were screened, and 189 were excluded owing to incomplete data. Eventually, 510 children, including 330 boys (64.71%) and 180 girls (35.29%), were included from the 19 hospitals. The children were divided into groups A (0.10 mg/kg/week ≤ PEG-rhGH < 0.15 mg/kg/week), B (0.15 mg/kg/week ≤ PEG-rhGH < 0.17 mg/kg/week), C (0.17 mg/kg/week ≤ PEG-rhGH < 0.20 mg/kg/week), and D (PEG-rhGH 0.20 mg/kg/week) according to the initial dosage. The baseline characteristics of the children are shown in Table 1. The children in group D were younger than those in groups A and C (*P* = 0.033 and *P* = 0.016, respectively), while there were no significant differences between groups B and D (*P* = 0.103). The children in group D had lower baseline BA/CA than those in group C (LSM difference [95%CI], -0.04, [-0.07, -0.01], *P* < 0.001), and higher BA/CA than those in group B (LSM difference [95%CI], 0.04 [-0.02, 0.07], *P* = 0.002), while there were no significant differences between groups A and B (LSM difference [95%CI], 0.02 [-0.03, 0.06], *P* = 0.432) and between groups A and D (LSM difference [95%CI], -0.03 [-0.07 ~ 0.01] *P* = 0.192). According to MRI, 25 (4.9%) patients were suspected of having pituitary stalk occlusion syndrome. All others had a normal brain MRI. Among the 510 children, 171 (33.5%) had a GH peak < 5 µg/L (the median GH peak was 6.11 (4.35–7.72) µg/l). In addition, 35 (6.9%) were previously treated with GH. Table 1 shows the distribution of IGF-1 SDS and GH peaks per dose group.

**Table 1:** Baseline characteristics of the patients

Characteristics	Total (*n* = 510)	A (*n* = 47)	B (*n* = 129)	C (*n* = 121)	D (*n* = 213)	*P* value
Age^a^, year, mean ± SD	8.03 ± 2.67	8.57 ± 3.24	8.13 ± 2.63	8.38 ± 2.34	7.65 ± 2.69	0.036
Sex, N (%)						0.191
Male	330 (64.71)	34 (72.34)	89 (68.99)	80 (66.12)	127 (59.62)	
Female	180 (35.29)	13 (27.66)	40 (31.01)	41 (33.88)	86 (40.38)	
Ht SDS, mean ± SD	-2.69 ± 0.80	-2.77 ± 1.13	-2.73 ± 0.72	-2.60 ± 0.59	-2.69 ± 0.87	0.530
Weight SDS, mean ± SD	-1.39 ± 0.65	-1.32 ± 0.87	-1.34 ± 0.65	-1.37 ± 0.58	-1.44 ± 0.64	0.429
BMI SDS, mean ± SD	-0.36 ± 1.09	-0.24 ± 1.19	-0.22 ± 1.11	-0.47 ± 1.16	-0.40 ± 1.03	0.245
BA, mean ± SD	5.75 ± 2.25	5.96 ± 2.58	5.55 ± 2.27	6.31 ± 1.96	5.50 ± 2.26	0.009
BA/CA, median (IQR)	0.72(0.63 ~ 0.80)	0.72(0.60 ~ 0.79)	0.68(0.58 ~ 0.77)	0.77(0.69 ~ 0.82)	0.72(0.63 ~ 0.80)	< .0001
IGF-1 SDS, median (IQR)	-0.93 (-1.42 ~ -0.43)	-0.88 (-1.38 ~ -0.37)	-1.00 (-1.48 ~ -0.43)	-0.99 (-1.38 ~ -0.65)	-0.87 (-1.39 ~ -0.35)	0.801
GH peak, µg/l, median (IQR)	6.11 (4.35 ~ 7.72)	5.70 (3.17 ~ 7.41)	5.66 (3.74 ~ 7.40)	6.86 (4.97 ~ 8.01)	6.17 (4.54 ~ 7.76)	0.004
GH peak, N (%)						0.023
< 5 µg/l	171 (33.53)	20 (42.55)	56 (43.41)	31 (25.62)	64 (30.05)	
5-7 µg/l	146 (28.63)	14 (29.79)	34 (26.36)	33 (27.27)	65 (30.52)	
> 7 µg/l	193 (37.84)	13 (27.66)	39 (30.23)	57 (47.11)	84 (39.44)	
Pituitary gland MRI l, N (%)						0.047
Normal	485 (95.10)	42 (89.36)	122 (94.57)	120 (99.17)	201 (94.37)	
Suspected Pituitary stalk blocking syndrome	25 (4.90)	5 (10.64)	7 (5.43)	1 (0.83)	12 (5.63)	
Previously treated with GH, N (%)	35 (6.86)	3 (6.38)	6 (4.65)	5 (4.13)	21 (9.86)	0.145

^a^Baseline age = (date of signing informed consent – date of birth + 1)/365.25; the results are described with two decimals

*SD* standard deviation, *Ht* height, *SDS* standard deviation score, *BMI* body mass index, *BA* bone age, *IGF-1* insulin-like growth factor-1, *GH* growth hormone

### Effectiveness and multivariate analysis

**Fig. 1:**
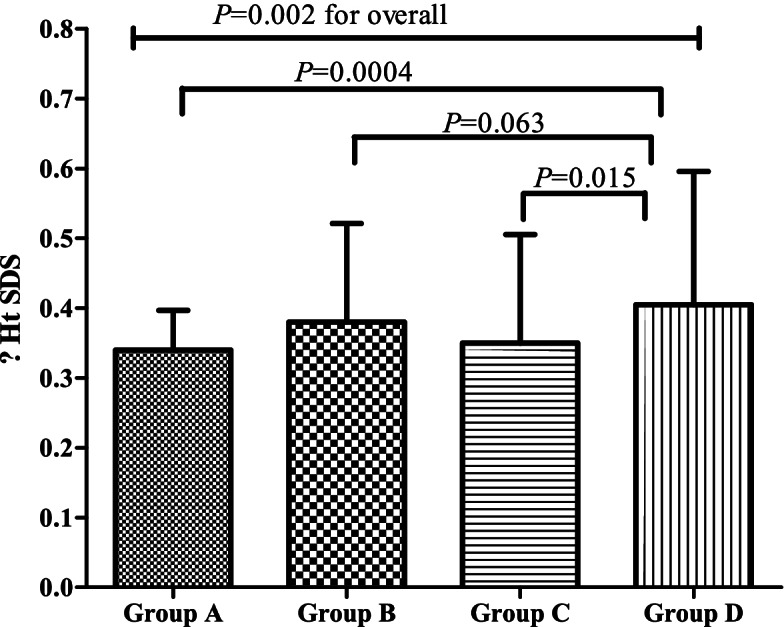
The distribution of 6-month ΔHt SDS in different groups

The 6-month ΔHt SDS for the total cohort was 0.49 ± 0.27, and the meanswere different among the four groups (*P* = 0.002). The best therapeutic effect was observed in group D (0.54 ± 0.27), which was higher than in group A (0.38 ± 0.30) (*P* = 0.0004 and *P* = 0.015, respectively). The 6-month ΔHt SDS of groups B (0.48 ± 0.28) and C (0.46 ± 0.24) was numerically higher than in group A (Fig. 1).

The covariance analysis showed that dosage was associated with the 6-month ΔHt SDS (*P* = 0.01) after adjusting for baseline covariates. The 6-month ΔHt SDS of group A was lower than in groups B (LSM difference [95%CI], -0.09 [-0.17, -0.01]), C (LSM difference [95%CI], -0.10 [-0.18, -0.02]), and D (LSM difference [95%CI], -0.13 [-0.21, -0.05]). No difference was observed among groups B, C, and D (Table 2).

**Table 2:** The covariance analysis of dosage group and 6-month ΔHt SDS

Covariance	*F*-value	*P*-value
Baseline IGF-1 SDS group	13.38	< .0001
Dosage group	3.85	0.0096
Baseline age	24.64	< .0001
Baseline Ht SDS	1.69	0.1948
Baseline BA	0.25	0.6142
Baseline BMI SDS	3.64	0.0568
Gender	5.2	0.0230
GH peak	1.28	0.2586
The 6-month ΔHt SDS according to dosage		
LSM (95% CI) in group A	0.43 (0.36, 0.51)	
LSM (95% CI) in group B	0.52 (0.46, 0.57)	
LSM (95% CI) in group C	0.53 (0.47, 0.58)	
LSM (95% CI) in group D	0.56 (0.52, 0.60)	
(A-B) LSM Difference (95%CI)	-0.09 (-0.17, -0.01)	
(A-C) LSM Difference (95%CI)	-0.10 (-0.18, -0.02)	
(A-D) LSM Difference (95%CI)	-0.13 (-0.21, -0.05)	
(B-C) LSM Difference (95%CI)	-0.01 (-0.07, 0.05)	
(B-D) LSM Difference (95%CI)	-0.04 (-0.10, 0.01)	
(C-D) LSM Difference (95%CI)	-0.03 (-0.09, 0.02)	

*Ht* height, *SDS* standard deviation score, *IGF-1* insulin-like growth factor-1, *BA* bone age, *BMI* body mass index, *GH* growth hormone, *LSM* Least-squares mean, *CI* confidence interval

The multivariable analysis showed that PEG-rhGH dosage (β = 2.173, 95%CI: 1.250 ~ 3.095, *P* < 0.001) was positively associated with the 6-month ΔHt SDS; baseline BA/CA (β = -0.285, 95%CI: -0.478, -0.092, *P* = 0.004) and baseline IGF-1 SDS (β = -0.031, 95%CI: -0.060, -0.002, *P* = 0.038) were negatively associated with the 6-month ΔHt SDS. Baseline Ht SDS (*P* = 0.768), baseline BMI SDS (*P* = 0.066), female (*P* = 0.828), and GH peak (*P* = -0.008) were not associated with the 6-month ΔHt SDS (Table 3).

**Table 3:** The multivariate regression analysis of prognostic factors of 6-month ΔHt SDS

Variable	β (95% CI)	P
Baseline BA/ Baseline CA	-0.285 (-0.478, -0.092)	0.0039
Baseline Ht SDS	-0.0045 (-0.038, 0.028)	0.7677
Dosage	2.173 (1.250, 3.095)	< 0.0001
Baseline IGF-1 SDS	-0.031 (-0.060, -0.002)	0.0378
Baseline BMI SDS	0.021 (-0.001, 0.043)	0.0655
Female	0.005 (-0.044, 0.055)	0.8281
GH peak	-0.008 (-0.018, 0.002)	0.1230

*Ht* height, *SDS* standard deviation score, *CI* confidence interval, *BA* bone age, *CA* chronological age, *IGF-1* insulin growth factor-1, *BMI* body mass index, *GH* growth hormone

### Subgroup analysis

**Fig. 2:**
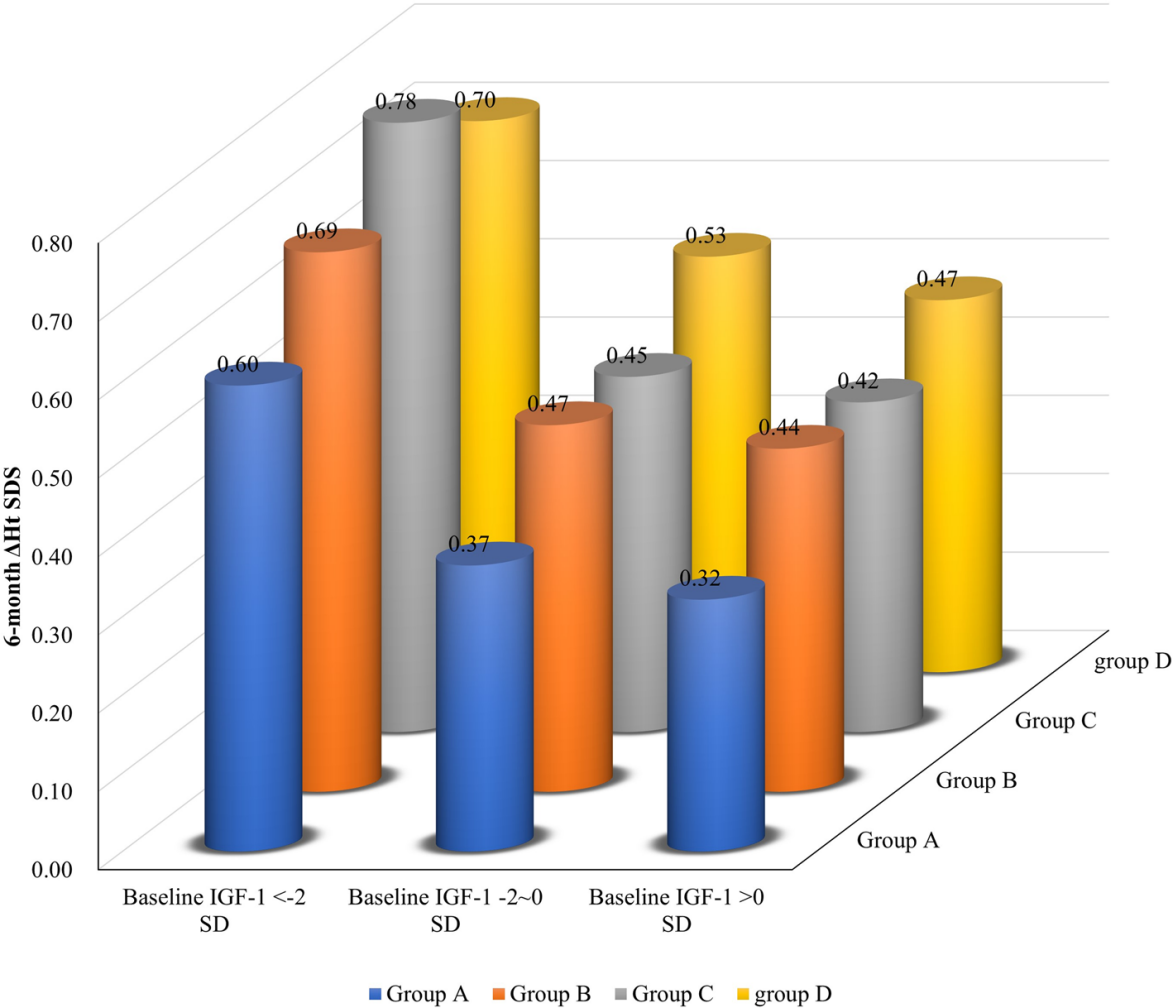
The 6-month ΔHt SDS distribution in different baseline IGF-1 SDS combined with different treatment dosages

The interaction analysis between baseline IGF-1 SDS and dosage showed that the baseline IGF-1 interacts with dosage (Fig. 2 and Table S1). The 6-month ΔHt SDS was not significantly different with different dosages among children with baseline IGF-1 < -2 SDS (*P* = 0.931), suggesting that the height gain was not significantly associated with the PEG-rhGH dosage. When the baseline IGF-1 SDS was between -2 SDS and 0 SDS, differences among the dosage groups were observed (*P* = 0.003). The dosages were linearly correlated with the treatment effectiveness, with higher dosages exerting higher height gains. When the baseline IGF-1 was > 0 SDS, the ΔHt SDS was not significantly different among the dosage groups (*P* = 0.398) (Table S1).

In the patients with IGF-1 SDS between -2 SDS and 0 SDS, subgroup analysis by baseline age subgroups (< 7, 7–9, and > 9) showed that ΔHt SDS was negatively correlated with age. The correlation coefficient Rho was -0.5 (*P* < 0.001), suggesting that the increase in Ht SDS was lower after 6 months of treatment in older children. In addition, the dosage was weakly correlated with age. The correlation coefficient Rho was -0.11 (*P* = 0.022), suggesting that the treatment dosage was less changed in older children and that the dosage tended to decrease gradually.

### Safety

The overall safety was good, and the treatment was tolerable. The incidence rates of treatment drug-related adverse responses (ADR) were 4/47 (8.51%), 11/129 (8.53%), 17/121 (14.05%), and 26/213 (12.21%) in groups A, B, C, and D, respectively. No severe adverse events (SAEs) were observed in group A. Two patients (2/129, 1.55%) in group B had SAEs, including head trauma and pneumonia. One patient (1/121, 0.83%) in group C had enteritis. No SAEs occurred in group D. Overall, two patients developed peripheral edema. No lipodystrophy was observed.

## Discussion

This study aimed to evaluate the effectiveness of individualized-dose PEG-rhGH treatment in children with short stature in a multicenter, large-scale, prospective, real-world study. We tried to examine the dosage for the initial PEG-rhGH application. PEG-rhGH is effective with different dosages ranging from 0.10 mg/kg/week to 0.20 mg/kg/week for children with short stature. The baseline factors such as BA/CA, baseline IGF-1 SDS, and dosage affect the 6-month ΔHt SDS, while the baseline of Ht SDS and BMI SDS, sex, and the GH peak did not. The previous phase 3 trials showed the efficacy of PEG-rhGH over short-acting GH for the management of GHD. Indeed, the phase 3 trial by Luo et al. [19] was performed in 343 children and showed that the ΔHt SDS was 0.90 ± 0.36 for the 0.10 mg/kg/week dosage and 1.01 ± 0.39 for the 0.20 mg/kg/week dosage, significantly different after 25 weeks. They also showed that children with peak GH levels ≤ 5 µg/L (severe GHD) had the largest changes in growth parameters compared with those with peak GH > 5 µg/L, but they analyzed the two dosages together. In a non-randomized study by Qiao et al. [9] et al., the Ht SDS increased from -2.57 ± 0.75 at baseline to -1.06 ± 0.85 at 24 months in children treated with PEG-rhGH. Other long-acting rhGH products showed similar results [12, 24, 25]. In the present study, the 6-month ΔHt SDS was 0.49 ± 0.27, which is a smaller improvement than previously reported. The 6-month ΔHt SDS increased with dosage, at 0.38 ± 0.30 for the 0.10–0.14 mg/kg/week dosage and 0.54 ± 0.27 for the 0.20 mg/kg/week dosage, and with intermediary values for the 0.15–0.16 and 0.17–0.19 mg/kg/week dosages. This study was a real-world study of the treatment of prepubertal slow-growing short children. The inclusion criteria for the short stature of children were not as strict as GHD patients in the phase 3 trial. The height increase at 6 months was smaller in the present study than in the phase 3 trials [12, 19, 25], which could be attributed to the stricter selection criteria in the phase 3 trials, while the present study included actual patients from routine clinical practices. The differences in patient selection led to differences such as lower GH peaks and lower median IGF-1 levels, which probably affected the growth outcomes. In addition, the starting dosage was not the same as in the phase 3 trials since it was based on the physicians’ clinical experience.

The hypothalamus-GH-IGF-1 axis is directly associated with growth [26], and IGF-1 is a commonly used marker of GH activity [25, 26]. The stratified analysis by baseline IGF-1 showed no dosage-dependent relationship between the 0.10 and 0.20 mg/kg/w dosages in the subgroups of baseline IGF-1 < -2 SDS and baseline IGF-1 > 0 SDS. It suggested that the height gain was not significantly associated with the PEG-rhGH dosage. Based on the baseline IGF-1 < -2 SDS, the clinical diagnosis is more likely to be GHD. The low initial dosage (0.10 mg/kg/week) could achieve a therapeutic effect. The number of patients with IGF-1 < -2 SDS was small, and studies with larger numbers are needed. Patients with baseline IGF-1 > 0 SDS were most likely not diagnosed with GHD in clinical practice. Although there were no statistically significant differences between the dose groups, the therapeutic effect of group A was numerically smaller than in the other groups. A dose of more than 0.20 mg/kg/week might be required to achieve a short-term therapeutic effect. Therefore, an initial dosage of 0.20 mg/kg/week might be recommended for patients with baseline IGF-1 > 0 SDS, while the subgroup of baseline IGF-1 of -2 ~ 0 SDS showed a significant association between treatment effectiveness and dosage. In addition, children with a younger age had better treatment effectiveness, suggesting that the treatment should be applied as early as possible. Ying et al. [8] showed that the factors affecting growth velocity were age at rhGH initiation, IGF-1 SDS during treatment and growth velocity in the year before treatment. Nevertheless, the subgroups in the present study were small and uneven in size. Future studies are necessary to confirm the results.

In addition, the children in group A were older than in the other groups. Due to the experience of clinicians or the results of the phase 2 trial, many clinicians believed that it is safer with a lower initial dosage. The age might be an important confounding factor in this study.

The present study did not report new safety signals compared with the previous phase 3 trial [19]. In the present study, no patient discontinued therapy due to ADRs. Still, SAEs did occur, including head trauma, pneumonia, enteritis, benign intracranial hypertension, upper respiratory infection, myocardial reperfusion injury, and tonsillitis. Of note, no SAEs were reported in the phase 3 trial [19], which might be because of patient selection. In the present study, no hypothyroidism [27], injection site lipoatrophy [27], or anti-rhGH antibodies [12, 25] were observed.

This study has some limitations. First, the follow-up is short, and long-term clinical outcomes are lacking. Second, dividing the patients into four dosage groups resulted in a small sample size of the 0.10–0.14 mg/kg/week group. Third, the sample size of the baseline IGF-1 < -2 SDS subgroup was small, and the results should be interpreted with caution. Further studies are needed to validate the findings in these children.

## Conclusions

In conclusion, PEG-rhGH is effective for children with short stature, with dosages ranging from 0.10 mg/kg/week to 0.20 mg/kg/week. The baseline IGF-1 interacts with dosage: for children with baseline IGF-1 of < -2 SDS, 0.10 mg/kg/week as an initial dose could be effective; when the baseline IGF-1 was ≥ -2SDS, initiating at a higher dosage with 0.15–0.20 mg/kg/week might be the better.

## Supplementary Information

**Additional file 1: Table S1.** Thedescription of 6-monthΔHt SDS among childrenwith different baseline characteristics and different treatment dosage

## Data Availability

The datasets used and/or analyzed during the current study are available from the corresponding author on reasonable request.

## Abbreviations

PEG-rhGH: Polyethylene glycol recombinant human growth hormone
Ht SDS: Height standard deviation score
ISS: Idiopathic short stature
GHD: Growth hormone deficiency
GH: Growth hormone
rhGH: Recombinant human growth hormone
GV: Growth velocity
BA: Bone age
ALT: Alanine transaminase
ULN: Upper limit of normal
Cr: Creatinine
ACTH: Adrenocorticotropic hormone
IGF-1: Insulin-like growth factors-1
IGFBP-3: Insulin-like growth factor binding protein-3
IGF-1: SDS IGF-1 standard deviation score
ANOVA: Analysis of variance
LSD: Least significant difference
ADR: Adverse responses
