# Supplementary material for: Polyethylene glycol recombinant human growth hormone in Chinese prepubertal slow-growing short children: doses reported in a multicenter real-world study
Source: BMC Endocr Disord. 2022 Aug 9;22:201. doi: 10.1186/s12902-022-01101-8 (PMC9364582; doi:10.1186/s12902-022-01101-8)
Supplement: Supplementary file 1 — Additional file 1: Table S1. Thedescription of 6-monthΔHt SDS among childrenwith different baseline characteristics and different treatment dosage [file 12902_2022_1101_MOESM1_ESM.docx]

Table S1. The description of 6-monthΔHt SDS among children with different baseline characteristics and different treatment dosage

|  | **Group A** | | **Group B** | | **Group C** | | **Group D** | | ***P*-value** |
| --- | --- | --- | --- | --- | --- | --- | --- | --- | --- |
| **Baseline IGF-1** | N | Mean (SD) | N | Mean (SD) | N | Mean (SD) | N | Mean (SD) |  |
| <-2 SDS | 4 | 0.60 (0.27) | 9 | 0.69 (0.31) | 4 | 0.78 (0.38) | 16 | 0.70(0.46) | 0.9312 |
| -2~0 SDS | 36 | 0.37 (0.31) | 102 | 0.47 (0.28) | 103 | 0.45 (0.23) | 167 | 0.53(0.26) | 0.0030 |
| >0 SDS | 7 | 0.32 (0.26) | 18 | 0.44 (0.27) | 14 | 0.42 (0.24) | 30 | 0.47(0.15) | 0.3987 |
